# A Potential Gut–Retina Axis in Retinopathy of Prematurity: Emerging Perspectives on Microbiome-Mediated Modulation of the IGF-1–VEGF Pathway

**DOI:** 10.3390/ijms27073317

**Published:** 2026-04-07

**Authors:** Shohan Shetty, Robert Luca, Sarah Hilkert Rodriguez, Dimitra Skondra

**Affiliations:** 1 Pritzker School of Medicine, University of Chicago, Chicago, IL 60637, USArobert.luca@uchicagomedicine.org (R.L.); 2 Department of Ophthalmology and Visual Science, University of Chicago, Chicago, IL 60637, USA; 3 Department of Ophthalmology, NYU Grossman School of Medicine, New York, NY 10016, USA

**Keywords:** retinopathy of prematurity, gut microbiome, IGF-1–VEGF axis, retinal angiogenesis, hypoxia-inducible factor-1α, neonatal inflammation, short-chain fatty acids, bile acids, oxygen-induced retinopathy, metabolomics

## Abstract

Retinopathy of prematurity (ROP) is a leading cause of childhood blindness characterized by disrupted physiologic vascularization followed by pathologic neovascularization, classically organized around the insulin-like growth factor-1 (IGF-1)–vascular endothelial growth factor (VEGF) axis in the retina. Increasing evidence suggests that early-life gut dysbiosis may act as an upstream modifier of this biphasic process. In this review, we synthesize human cohort studies, multi-omics analyses, and experimental animal models examining associations between the neonatal gut microbiome and ROP. Preterm infants who develop severe ROP demonstrate enrichment of facultative anaerobes and reduced acquisition of obligate anaerobes, alongside altered predicted metabolic capacity. Microbiome-derived metabolites, including short-chain fatty acids, bile acid derivatives, and lipid mediators, have been shown in experimental systems to influence systemic IGF-1 production, hypoxia-inducible factor-1α stabilization, and VEGF signaling. Rodent oxygen-induced retinopathy models offer a translation framework to assess the functional link between microbial perturbation and retinal angiogenic responses. Collectively, these findings support a conceptual microbiome–IGF-1–VEGF–retina axis in which early intestinal dysbiosis may modulate inflammatory tone, metabolic signaling, and retinal vascular development. Although current evidence remains largely associative, integrating microbiome profiling with mechanistic and longitudinal studies may clarify potential causal pathways and identify novel biomarkers or preventive strategies for severe ROP.

## 1. Introduction

Retinopathy of prematurity (ROP) is a retinal disease that primarily affects premature and very-low-birth-weight infants. Despite advances in neonatal care, ROP remains one of the leading causes of childhood blindness globally [1,2]. The pathogenesis of retinopathy of prematurity is best described as a biphasic process organized around the IGF-1–VEGF axis [3,4,5,6]. In phase I, preterm birth abruptly exposes the developing retina to relative hyperoxia, suppressing VEGF-mediated endothelial signaling at a time when circulating IGF-1 is already critically low due to loss of placental supply and systemic immaturity [3]. Together, these deficits impair orderly peripheral retinal vascularization, producing areas of avascular retina. In phase II, the metabolic demands of the growing but incompletely vascularized retina stabilize hypoxia-inducible factor-1α (HIF-1α), which drives VEGF upregulation. As endogenous IGF-1 recovers postnatally, it permits and amplifies VEGF signaling; however, when IGF-1 recovery is delayed or insufficient, this response becomes dysregulated, promoting pathologic neovascularization, which is prone to complications and can progress to retinal detachment and blindness if untreated [4,5].

A recent nationwide cohort study analyzing over 23 million births across almost 2 decades demonstrated that the proportion of premature infants diagnosed with ROP nearly doubled in the United States, rising from 4.4% to 8.1% [6]. This burden of disease is not equally distributed. Black and Hispanic infants experienced the most dramatic increases, with ROP incidence reaching 11.6% and 8.2%, respectively, and infants from the lowest-income quartile and those born in the Southern United States carried the highest risk [6]. Although earlier gestational age and birth weight may explain some of these differences, these findings underscore that ROP disproportionately affects historically underserved populations [7]. These disparities likely reflect not only differences in prematurity, but also variation in early-life exposures—including antibiotic use, feeding practices, and neonatal intensive care environments—that are known to influence infant development and disease risk. Understanding novel biological pathways through which these exposures may contribute to ROP could help identify new opportunities for prevention and more equitable care, thus highlighting its importance.

Among these, the gut microbiome has emerged as a compelling candidate. In infants, the gut microbiota is shaped by early-life exposures such as antibiotic use, cesarean delivery, varying feeding practices, and NICU environment. Imbalances of these communities have been implicated in conditions such as necrotizing enterocolitis (NEC), bronchopulmonary dysplasia (BPD), and sepsis. Studies have also described a gut–retina axis, suggesting the intestinal microbiota as a modulator of retinal homeostasis and disease [8]. Dysbiosis has been linked to ocular conditions, including age-related macular degeneration, diabetic retinopathy, glaucoma, uveitis, and autoimmune dry eye, with accumulating evidence indicating that alterations in gut microbial composition, reduced diversity, and depletion of beneficial metabolite-producing bacteria can promote chronic systemic inflammation, increase gut permeability, and disrupt immune and metabolic homeostasis. These changes may lead to the release of pro-inflammatory cytokines, endotoxins, and altered microbial metabolites into circulation, which can impair the blood–retinal barrier, exacerbate oxidative stress, and drive pathological angiogenesis and neurodegeneration, thereby contributing to retinal degeneration, microvascular injury, neuroinflammation, and aberrant immune activation underlying both ocular surface disease and intraocular inflammation [7,8,9,10,11,12,13,14,15]. Emerging preclinical and clinical studies suggest that microbiome-mediated pathways, which include inflammatory modulation, metabolic signaling, and immune activation, might also influence retinal angiogenesis and ROP development [12,16]. 

**Figure 1:**
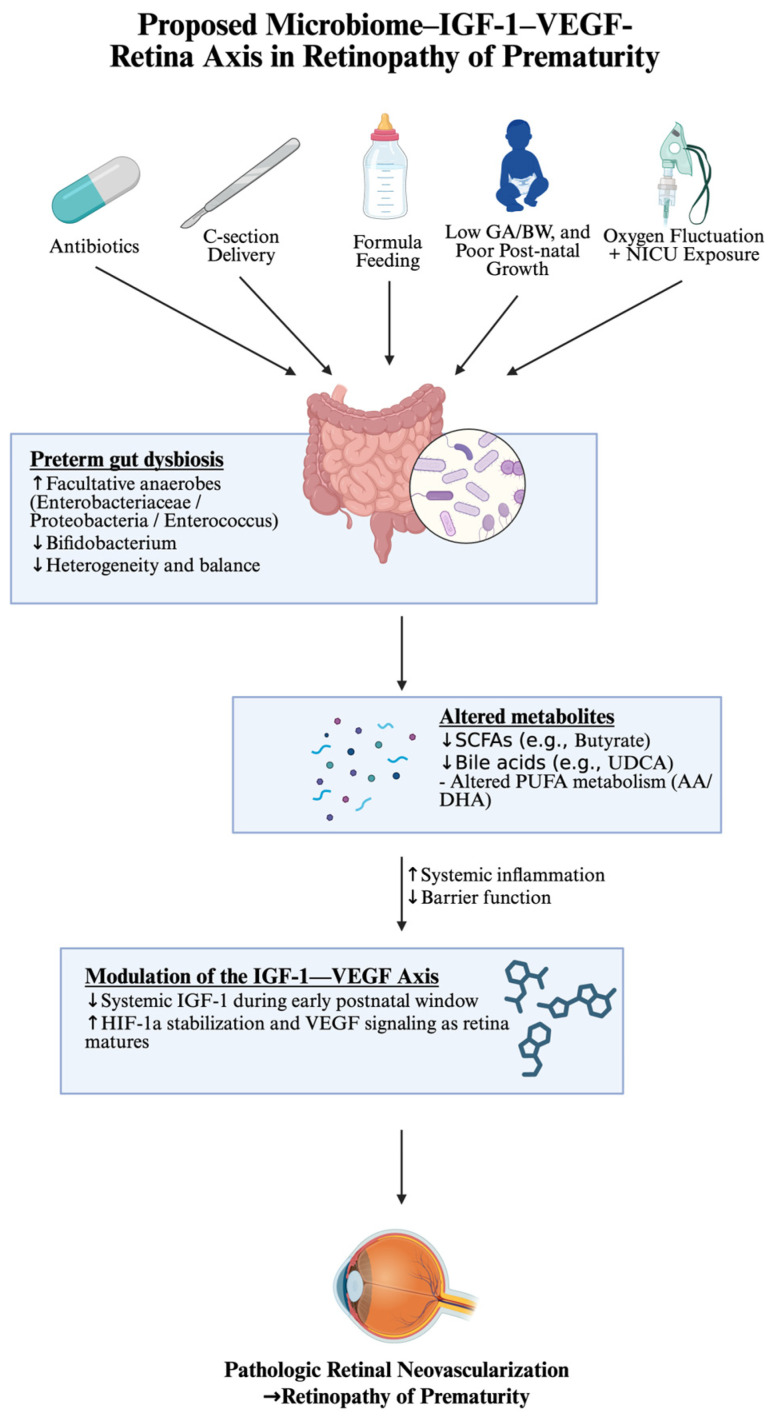
Proposed microbiome–IGF-1–VEGF–retina axis in retinopathy of prematurity. Schematic representation of hypothesized interactions linking early-life exposures (e.g., antibiotic use, delivery mode, feeding practices, and NICU environment) to preterm gut dysbiosis. Altered microbial composition and reduced diversity are associated with changes in metabolite production, including short-chain fatty acids, bile acid derivatives, and lipid mediators. These shifts may contribute to systemic inflammation, impaired barrier function, and modulation of the IGF-1–VEGF axis, characterized by early suppression of IGF-1 and subsequent dysregulated VEGF signaling. Together, these processes may influence retinal vascular development and promote pathologic neovascularization in retinopathy of prematurity.

This study aims to summarize the emerging literature, which suggests early-life intestinal dysbiosis may shape retinal vascular risk through inflammatory and metabolite-mediated effects on the IGF-1–VEGF axis (Figure 1). ROP is a complex and dynamic condition. The gut microbiome is unlikely to represent a singular or definitive cause of ROP; rather, it may constitute one modifiable upstream contributor within this broader biological landscape. Throughout this review, proposed mechanistic links between microbiome-derived metabolites and retinal angiogenesis are drawn from associative human data and experimental models. Unless otherwise stated, these connections should be understood as biologically plausible hypotheses rather than established causal pathways, and the strength and limitations of the underlying evidence are discussed where relevant.

## 2. The Development of the Newborn Microbiome

The establishment and development of the newborn’s gut microbiome is a dynamic process influenced by maternal, peripartum, and postnatal factors. While the literature is split between the “sterile womb paradigm” and the “in utero colonization hypothesis”, there is increasing evidence that microbial colonization can begin in utero [17]. Regardless, the most significant expansion and diversification of the gut microbiome occurs during and after birth. This is heavily influenced by maternal microbiota, mode of delivery, gestational age, and intrapartum antibiotic exposure. These risk factors, which predispose preterm infants to gut dysbiosis, overlap considerably with those that increase susceptibility to ROP [18,19,20,21,22,23,24].

In term infants, gut colonization typically follows an orderly succession. Early facultative anaerobes (*Enterobacteriaceae*, *Streptococcus*) give way to obligate anaerobes (*Bifidobacterium*, *Bacteroides*, *Clostridia*) over the first few weeks of life, supporting barrier maturation, immune tolerance, and metabolic homeostasis [25]. In contrast, preterm infants exhibit delayed anaerobe acquisition, reduced microbial richness and community balance (α-diversity), and instability with enrichment of opportunistic and hospital-acquired taxa (e.g., *Klebsiella*, *Enterococcus*, and *Staphylococcus*).

The mode of delivery also plays a central role in shaping the early gut microbiome [26]. Vaginal delivery facilitates the transfer of maternal vaginal and fecal microbes such as *Lactobacillus*, *Escherichia*, *Bacteroides*, *Bifidobacterium*, *Streptococcus*, and *Prevotella* [27]. Cesarean section is also associated with exposure to maternal microbiota, but initial colonization is most influenced by environmental isolates from equipment, air, and other infants, with hospital staff being the primary vectors for transference [27]. Limited research has been done to understand the difference in ROP rates between infants born through vaginal delivery compared with cesarean section, but a recent meta-analysis including 5 cohort studies involving 2048 babies found a higher incidence of ROP in infants born through vaginal delivery compared with cesarean section [20]. Although vaginal delivery is typically associated with more favorable early microbial colonization, this counterintuitive association may reflect non-microbial factors, including mechanical stress during labor, perinatal hypoxia, and residual confounding by gestational age, an independent and strong predictor of ROP [18,28,29,30,31]. Nevertheless, given the well-established differences in early gut microbial composition between delivery modes, microbiome-mediated mechanisms remain a plausible pathway for differences in ROP risk [32,33].

The mode of infant feeding has also been shown to be an important factor influencing the infant gut microbial colonization and composition [34]. While infant formula has been designed to mimic human milk as closely as possible, the gut microbiome of infants consuming formula milk differs from that of infants who receive human milk. Human milk contains live bacteria, human milk oligosaccharides, immunoglobulins, and other bioactive compounds that promote colonization by beneficial microbes and support immune development [35]. Breastfeeding has been shown to establish a neonatal gut microbiome dominated by *Bifidobacterium* and *Lactobacillus* with lower overall diversity and reduced abundance of taxa such as Enterobacteriaceae and Staphylococcus, compared to formula feeding [36,37]. It is associated with lower levels of *Ruminococcaceae*, *Lachnospiraceae*, and other taxa associated with inflammation and metabolic activity [38]. Feeding with breast milk has been associated with lower rates of gut dysbiosis, reduced systemic inflammation, and improved intestinal barrier function, all of which are implicated in the pathogenesis of ROP [39,40,41]. This is perhaps one mechanism by which ROP rates are decreased in breastfed newborns [21,42]. Several observational studies have attempted to account for confounding through multivariable adjustment for established clinical predictors of ROP [43,44]. However, because feeding type is not randomized, it may also reflect underlying clinical vulnerability; infants with slower postnatal growth or greater medical instability are more likely to require formula supplementation, raising the possibility of confounding.

Antibiotics are widely prescribed in preterm neonates [45]. Prolonged courses of these have the potential to decrease microbial diversity, suppress obligate anaerobes, and select for proteobacteria, with downstream risks for NEC, late-onset sepsis (LOS), and systemic inflammation [46,47]. In one retrospective study, early exposure to specific antibiotic classes, particularly cephalosporins, carbapenems, and monobactams, has recently been shown to be associated with ROP; while sicker infants are more likely to receive antibiotics, the broad-spectrum coverage of these particular classes may also contribute to their heightened risk [23]. LOS, which can be a downstream consequence of neonatal antibiotic exposure, has itself been associated with severe ROP [48,49,50]. Importantly, sepsis may also contribute indirectly to ROP by impairing postnatal growth trajectories, a robust predictor of severe ROP in weight-gain-based risk models [51,52]. Even when analyses adjust for sepsis, such adjustment may incompletely capture the broader constellation of clinical instability, inflammatory burden, and treatment exposures that characterize vulnerable infants and contribute to ROP risk.

Because the same exposures that sculpt the preterm microbiome (delivery, feeding, antibiotics, NICU stay, comorbid infection/NEC) are also ROP risk modifiers, the microbiome is plausibly a proximal mediator rather than a bystander. These overlapping determinants are summarized in Table 1. Studies increasingly show that microbial and metabolic signatures precede clinical pathologies of prematurity, supporting predictive and mechanistic relevance [53]. This positions the microbiome as a prime candidate for integration into risk stratification, biomarker discovery, and preventive interventions.

## 3. Microbial Changes Associated with ROP

Several groups have profiled stool from preterm infants who do and do not develop ROP using 16S rRNA sequencing. These studies are associative, but they allow for comparison of microbial community structure and inferred functional capacity between infants with divergent retinal outcomes. Across cohorts, recurring microbial patterns have emerged that suggest biologically plausible links between early intestinal ecology and retinal vascular risk [66,67,68,69].

### 3.1. Enrichment of Inflammation-Prone Facultative Anaerobes

Across cohorts, infants with treatment-requiring ROP more often show relative enrichment of facultative anaerobes and opportunistic taxa, particularly within *Proteobacteria*/*Enterobacteriaceae*, alongside inflammatory taxa commonly associated with the NICU environment (e.g., *Enterococcus*, *Klebsiella*, *Staphylococcus*) [66,68,69,70].

Early work from our group comparing preterm infants with type-1 (severe) ROP to matched high-risk controls without ROP identified significant enrichment of *Enterobacteriaceae* at 28 weeks postmenstrual age in infants who developed ROP [68]. Pathway analysis from the study suggests relative enrichment of amino acid metabolism and short-chain fatty acid-related pathways in infants without ROP, raising the possibility that early microbial composition influences systemic metabolic signaling relevant to vascular development. Overabundance of *Enterobacteriaceae* has also been associated with age-related macular degeneration, another VEGF-driven neovascular retinal disease, supporting the plausibility of shared inflammatory or metabolic mechanisms across retinal angiogenic disorders [71].

Whether *Enterobacteriaceae* enrichment represents a causal contributor to ROP pathogenesis or instead reflects upstream clinical exposures shared with ROP risk remains uncertain. Antibiotics, formula feeding, and NICU environmental exposures have all been linked to higher levels of *Enterobacteriaceae*, suggesting that this signal may, at least in part, mark a broader dysbiotic and inflammation-prone intestinal milieu [26,37,72].

### 3.2. Altered Acquisition of Putatively Protective Anaerobes and Sources of Heterogeneity

Additionally, infants who develop severe ROP have been reported to have reduced abundance of obligate anaerobes, particularly *Bifidobacterium*, suggesting that impaired early acquisition of these taxa may predispose to systemic inflammation and altered metabolic signaling [66]. *Bifidobacterium* plays a central role in shaping early gut ecology by promoting regulatory immune signaling pathways, dampening excessive inflammatory responses, and limiting the expansion of facultative opportunistic pathogens through competitive effects [73,74,75].

However, this association is not uniform across all studies. A recent multi-omics study integrating 16S sequencing with metabolomics identified higher relative abundance of *Bifidobacterium* in infants with ROP, alongside taxa such as *Staphylococcus* and *Klebsiella* [67]. However, the authors attributed this contradictory finding to probiotic supplementation given to many members of the ROP group, which was confirmed by further subgroup analysis. Differences in probiotic exposure, timing of sampling (early postnatal weeks versus later postmenstrual ages), antibiotic use, and NICU-specific practices likely influence much of the observed variability across studies and highlight the importance of contextualizing single-taxon findings.

### 3.3. Diversity Metrics and Inferred Functional Capacity

Beyond specific taxa, several studies have examined overall microbial community diversity as a potential marker of gut ecosystem stability. Alpha-diversity measures quantify the richness (number of taxa) and evenness (distribution of taxa) within a single sample and are often used as indicators of microbiome health or maturity. These measures have shown inconsistent associations with ROP, including reports of higher Chao, ACE, and Shannon indices with lower Simpson indices in infants with ROP [67]. Diversity metrics are highly sensitive to clinical exposures and developmental timing, limiting their interpretability as standalone biomarkers without standardized longitudinal sampling.

PICRUSt2 (Phylogenetic Investigation of Communities by Reconstruction of Unobserved States) is a computational tool that predicts the metabolic pathways encoded by a microbial community based on 16S rRNA sequencing data rather than through direct measurements of gene expression or metabolite production. Using this approach, ROP-associated communities have been predicted to be enriched in membrane transport, carbohydrate metabolism, and amino acid metabolism pathways, hinting that metabolic capacity is altered in the ROP-gut microbiome [66].

Collectively, these human microbiome studies associate severe ROP with a gut community structure characterized by enrichment of opportunistic facultative anaerobes, context-dependent alterations in early anaerobe acquisition, and metabolic differences. These associative patterns provide a rationale for mechanistic studies testing whether microbiome-derived metabolites and inflammatory signaling influence angiogenic pathways relevant to ROP.

## 4. Metabolomics and ROP

Recent human studies of retinal vascular disease demonstrate consistent gut microbial compositional shifts but are largely limited to taxonomic associations [12,76,77]. What remains less defined is how these microbial changes translate into angiogenic signaling. Microbial metabolites offer a more direct link between gut ecology and retinal biology than taxonomy alone. In preterm infants, shifts in community structure can translate into shifts in circulating metabolites that shape inflammation, endothelial signaling, and systemic growth pathways relevant to the IGF-1–VEGF timeline.

Short-chain fatty acids (SCFAs) are among the best-characterized microbial metabolites. They are produced as gut bacteria ferment dietary fiber in the intestines. SCFAs have roles inhibiting HDACs, promoting oligodendrocyte differentiation and myelination, and preserving blood–brain barrier integrity, among other things [26,78]. Preterm infants exhibit reduced gene abundance for SCFA synthesis [26]. In mice, antibiotic-mediated microbiome depletion reduces circulating IGF-1, and SCFA supplementation can partially restore IGF-1 levels, supporting a metabolite-to-endocrine signaling link [79]. This pathway may be relevant to ROP as IGF-1 is a key regulator of physiologic retinal vascular growth; insufficient IGF-1 delays this vascularization, expanding areas of avascular retina that later drive hypoxia-induced VEGF upregulation. Reduced SCFA production could therefore contribute to prolonged vulnerability during this developmental window.

A recent study found that butyrate, one of the gut’s most abundant SCFAs, is significantly reduced in a mouse model of ROP and that treatment with oral butyrate improved the ROP-relevant pathology [80]. A follow-up study suggests that this effect is mediated by butyrate’s role in suppressing HIF-1α and VEGF expression via increased H3 acetylation and decreased histone deacetylase activity [81]. Given that HIF-1α is the oxygen-sensitive transcription factor that drives VEGF expression during the hypoxic phase of ROP, modulation of this pathway provides a possible mechanistic link between gut-derived metabolites and retinal angiogenic signaling.

Secondary bile acids represent another microbially generated metabolite class with relevance to ROP. Ursodeoxycholic acid (UDCA), formed from primary bile acids through bacterial action in the intestines, reduced neovascularization in oxygen-induced retinopathy models [82]. The bile acid prevented reactive gliosis, preserved ganglion cell survival, and decreased OIR-induced blood retinal barrier dysfunction, all key features of retinopathy of prematurity. UDCA also decreased VEGF expression in the OIR retinas and prevented VEGF’s pro-angiogenic and pro-permeability effects. These findings suggest that bile acid-mediated modulation of VEGF signaling may be particularly relevant during Phase II ROP, when hypoxia-induced HIF-1α stabilization drives pathologic VEGF upregulation.

Microbial activity also shapes the availability and downstream metabolism of host lipids implicated in ROP. The Guo study found that linoleic acid, a polyunsaturated omega-6 fatty acid, was significantly elevated in infants who developed ROP. Linoleic acid can shape angiogenic tone through lipid signaling pathways that intersect with VEGF [83]. While linoleic acid itself is not produced by the gut microbiome, microbial effects on bile acid composition, intestinal absorption, and lipid metabolism may modulate its downstream pathways via chemical transformation into a more bioactive form and regulate how much the body can use [84].

Two of these downstream products, arachidonic acid (AA) and docosahexaenoic acid (DHA), are long-chain polyunsaturated fatty acids (PUFAs) whose metabolism is strongly shaped by gut microbial activity. A recent randomized clinical trial found that enteral supplementation with AA and DHA resulted in a 50% reduction in severe ROP in premature infants [85]. Their effects are possibly mediated by anti-inflammatory properties and inhibition of angiogenesis by the oxidized metabolite 4-hydroxy-docosahexanoic acid [86].

Several studies also point to disruption of amino acid and signaling metabolite pathways.

One analysis found that creatine was downregulated in the plasma of ROP patients, while levels of citrulline, arginine, and aminoadipic acid were upregulated. Pathway analysis of this study revealed biosynthesis of amino acids and the metabolism of arginine and proline to be significantly affected in ROP [87]. Gut microbiota play a central role in shaping host amino acid availability through direct synthesis, degradation, and modulation of intestinal absorption, and shifts in microbial composition can alter circulating levels of these metabolites [88,89,90]. Given the importance of arginine-derived nitric oxide in vascular tone and angiogenesis, these findings further support the presence of a metabolically primed pro-angiogenic environment.

These metabolites and the pathways that they influence yield insights into the mechanisms by which the gut microbiome might influence ROP development. Beyond advancing pathophysiologic understanding, these metabolites hold promise as biomarkers for identifying high-risk infants and as potential therapeutic targets to modulate disease progression. Given these metabolite-driven mechanisms, animal models provide an essential platform to establish causality between microbial shifts, metabolic outputs, and the retinal vascular response in ROP.

## 5. The IGF-1–VEGF Axis

IGF-1 and VEGF act in a time-dependent partnership during retinal vascular development, and disruption of their normal trajectories is strongly associated with severe ROP [91]. The gut microbiome has been linked to modulation of both of these integral molecules, thus highlighting the potential role of gut dysbiosis as a plausible upstream modifier of early IGF-1 trajectories and subsequent VEGF-driven neovascularization.

### 5.1. IGF as Systemic Growth Support for Retinal Vascularization

In utero, IGF-1 is maintained via the placenta and rises with gestational age, supporting VEGF-dependent endothelial survival and steady intraretinal vessel growth [85]. After preterm birth, loss of placental supply, suboptimal nutrition, and critical illness cause a sharp fall in serum IGF-1; in these instances, premature infants often remain far below term in utero levels for weeks [92]. Low IGF-1 during this early postnatal window is consistently associated with delayed physiologic vascularization and increased risk of ROP, making IGF-1 a plausible point where systemic exposures, including gut-derived signals, can influence retinal outcomes.

Large prospective cohort studies demonstrate that poor early postnatal weight gain strongly predicts severe ROP [93]. Because circulating IGF-1 levels closely track somatic growth in premature infants, early weight gain has been widely adopted as a clinical proxy for systemic IGF-1 exposure [55,94]. These findings reinforce the concept that disrupted postnatal growth trajectories, a clinical proxy for IGF-1 suppression, contribute directly to impaired physiologic vascularization and later proliferative disease. Importantly, poor postnatal growth in premature infants is frequently linked to systemic inflammation, sepsis, and antibiotic exposure—all of which are associated with gut dysbiosis [95,96]. While causality is not certain, this overlap raises the possibility that microbiome disruption represents one upstream contributor to the inflammatory and metabolic conditions that shape early IGF-1 trajectories.

### 5.2. Timing of Hypoxia/VEGF Signaling Relative to IGF-1 Recovery

Within the established biphasic model, VEGF accumulates during the avascular hypoxic phase and drives neovascularization once IGF-1 recovers. Cohort data suggest that in infants who progress to proliferative ROP, IGF-1 remains suppressed longer and then rises around the postmenstrual age when neovascular ROP typically manifests, consistent with a temporal coupling in which hypoxia-driven VEGF builds during the avascular period and clinically apparent pathologic angiogenesis emerges when IGF-1 recovers enough to support a strong endothelial response [97]. This timing is important for microbiome research because the gut ecosystem can influence factors that plausibly prolong or shorten IGF-1 deficiency during the early postnatal window.

### 5.3. Translational Touchpoints

Biomarker studies reinforce this framework, with low early IGF-1 and elevated VEGF among the strongest molecular correlates of ROP progression [98]. Clinically, anti-VEGF therapies act on the downstream limb of this axis, whereas upstream strategies that optimize growth and systemic signaling may modify risk earlier in the disease course [99].

### 5.4. How the Gut Microbiome May Modulate the IGF-1-VEGF Axis

#### 5.4.1. Dysbiosis-Linked Inflammation and Barrier Function

Emerging evidence suggests that the gut microbiome may represent an upstream regulator of the IGF-1-VEGF axis, adding a systemic layer to ROP susceptibility [16,100]. The gut dysbiosis seen in preterm infants is associated with impaired barrier function, systemic inflammation, and metabolic shifts relevant to vascular biology [101,102]. In this context, dysbiosis may act less like a direct trigger and more as a modifier of systemic growth and inflammatory tone that shapes IGF-1 trajectories and retinal vulnerability to hypoxia-driven angiogenic signaling.

#### 5.4.2. Microbial Metabolites as Upstream Signals

Microbial metabolites such as short-chain fatty acids (SCFAs) and bile acid derivatives influence hepatic and intestinal IGF-1 production, which in turn modulates peripheral angiogenic balance [103]. As detailed in Section 4, short-chain fatty acids may support systemic IGF-1 production and suppress hypoxia-driven HIF-1α stabilization, whereas bile acid derivatives such as UDCA have been shown to attenuate VEGF expression and neovascularization in experimental models. Together, these metabolites may influence both the permissive IGF-1 arm and the angiogenic VEGF arm of the ROP axis, suggesting roles in shaping the balance between physiologic vascularization and pathologic neovascularization rather than acting exclusively within a single disease phase.

Dysbiosis in premature infants, driven by antibiotic exposure, delayed enteral feeding, sepsis, or limited microbial diversity, has been linked with lower circulating IGF-1 and altered inflammatory signaling, potentially amplifying early vascular arrest and heightening retinal sensitivity to hypoxia [48,49,50,104]. This gut–endocrine–retina axis suggests that microbial composition and metabolite profiles could modify not only systemic growth signaling but also local angiogenic tone, which may potentially position the microbiome as a novel and potentially modifiable factor in ROP prevention and therapy if confirmed [77].

A useful working hypothesis is that early dysbiosis shifts inflammatory tone and metabolite availability, contributing to lower IGF-1 during the window of physiologic vascularization, enlarging the avascular retina and strengthening hypoxia/VEGF signaling. Animal models are then the place to test whether perturbing the microbiome during this window causally shifts IGF-1 trajectories and retinal vascular outcomes.

## 6. Animal Models

Animal models provide a critical bridge between associative human microbiome studies and causal inference. By allowing controlled manipulation of oxygen exposure, microbial composition, and host metabolism, experimental models make it possible to directly test whether gut dysbiosis and its downstream metabolites can modify retinal vascular development and neovascular responses relevant to ROP.

Most experimental work in this area relies on rodent oxygen-induced retinopathy (OIR) paradigms, with murine models serving as the primary platform and rat models offering complementary insight into peripheral vascular arrest and postnatal growth-related features of disease [82,105,106,107,108,109,110,111,112]. Importantly, these models can be combined with germ-free conditions, antibiotic-mediated dysbiosis, or defined microbial reconstitution, enabling targeted perturbation of the gut microbiome during critical developmental windows [79,113,114,115,116]. Additional species, such as felines, canines, and zebrafish, support specialized mechanistic and translational investigations that either cannot be fully addressed in mouse or rat paradigms or are more efficiently done in other animals [112,117,118].

In this setting, animal models function primarily as experimental platforms for testing whether microbiome-associated inflammatory and metabolic signals are sufficient to alter angiogenic balance in the developing retina. When interpreted alongside human data, these studies strengthen the biological plausibility of a gut–retina axis contributing to ROP pathogenesis.

## 7. Therapeutic Implications and Future Directions

If gut dysbiosis contributes to ROP, then altering the newborn microbiome—potentially via a combination of prebiotics, postbiotics, dietary supplementation, and/or fecal microbiota transplants—might offer preventive or therapeutic benefits. Antibiotics undoubtedly save lives, but given that antibiotics strongly disrupt the preterm gut flora, care teams should minimize unnecessary antibiotic exposure and consider more narrow-spectrum options rather than empiric broad therapy when possible [72]. Continued research should be done to understand how specific antibiotic regimens directly influence ROP [119]. Encouragingly, gut diversity tends to recover when antibiotics are discontinued, especially if infants receive mothers’ milk [120].

While a prior meta-analysis had shed doubt on the effectiveness of probiotics in reducing the risk of ROP, it was based on studies that were primarily looking at other outcomes and had ROP as a secondary result [121]. One recent study focused on ROP found that probiotic use was associated with a reduced risk in preterm, low-birth-weight infants, possibly by rebalancing the gut ecosystem [122]. However, probiotic supplementation may not fully recapitulate the ecological and developmental effects of naturally acquired bacterial colonization. Most probiotic strategies involve administration of a limited number of strains and may result in transient colonization without establishing the broader network and signaling dynamics. This may partially explain why probiotic exposure has not consistently translated to reduced ROP incidence despite observational links between dysbiosis and retinal vascular outcomes. Given their relative accessibility and biologic plausibility, more research should be done to understand whether they might play a role in ROP prophylaxis.

Importantly, the proposed microbiome–IGF-1–VEGF–retina axis should be viewed as a conceptual framework rather than a validated mechanistic pathway. Much of the existing evidence is derived from observational human studies and experimental models, each with inherent limitations. Microbial associations may reflect upstream clinical exposures such as antibiotic use, illness severity, or feeding practices, rather than direct contributors to disease. Additionally, many studies rely on 16S rRNA sequencing, which infers microbial function without directly measuring metabolite production or activity. Although animal models provide important mechanistic insight, they may not fully capture the complexity of human neonatal physiology. As such, current data support biological plausibility rather than causality.

Future work should focus on multi-center longitudinal cohorts with standardized sampling, integrated multi-omics approaches, and careful control of confounding clinical variables. Such efforts will be essential to determine whether microbiome alterations play a causal role in ROP pathogenesis or serve as biomarkers of disease risk. Advancing from association to mechanism, and ultimately to intervention, will require rigorous validation across both human and experimental systems. If these relationships are confirmed, targeted modulation of the neonatal gut microbiome could represent a novel strategy to reduce severe ROP and improve long-term ocular and systemic outcomes.

## Figures and Tables

**Table 1:** Clinical determinants of gut microbiome composition that overlap with risk factors for retinopathy of prematurity (ROP). Major risk factors for ROP—including lower gestational age and birth weight, poor postnatal growth, delivery mode, feeding practices, antibiotic exposure, and oxygen therapy—also shape early gut microbial development. These exposures are associated with reduced microbial diversity, enrichment of facultative anaerobes, delayed acquisition of beneficial taxa, and altered metabolic signaling. Collectively, these shared determinants support the gut microbiome as a potential intermediary linking early-life exposures to disrupted retinal vascular development.

Determinant	Microbiome Effect in Infant	ROP Association	Recent Evidence
GA/BW	Decreased gestational age decreased the relative abundance of *Bacteroidetes* while increasing that of *Proteobacteria*. Low gestational age infants exhibit significantly lower alpha diversity [18].	Every week decrease in GA and every 100 g decrease in BW led to a 1.4x and 1.8x increase, respectively, in the odds of developing ROP [28].	Kim et al., 2024 [18]
Poor post-natal growth	Poor postnatal growth is characterized by a dysbiotic microbial signature marked by low diversity and overabundance of potentially pathogenic strains and lack of beneficial anaerobes [54].	Poor postnatal growth and gut dysbiosis lead to low IGF-1 levels, stalling retinal vessel development and triggering ROP [55].	Li et al., 2025 [19]
Delivery mode (vaginal vs. C-section)	C-section newborns show delayed *Bacteroides*/*Bifidobacterium* acquisition, more skin/hospital taxa, and altered early community structure [19].	Limited data, but a recent meta-analysis found that vaginal delivery is associated with ROP, which aligns with early studies on the topic [20,30,56,57,58].	Sumual et al., 2024, Li et al., 2025, Zhou et al., 2023 [19,20,58]
Feeding (human milk vs formula)	Human milk provides live microbes, human milk oligosaccharides, immunoglobulins, and bioactives that enrich bifidobacteria and lactobacillus dominant communities; formula feeding yields higher proportions of *Enterococci* and *Clostridia* [22,40].	Predominant breast milk feeding has been found to reduce the risk of any stage ROP [1,21,59,60,61].	Peng et al., 2022, Notarbartolo et al., 2022, Goyal et al., 2024 [21,22,61]
Antibiotics	Leads to less diverse bacterial populations, decreases in bifidobacteria and *Bacteroidetes*, and increases in *Enterococcus* [24].	Early exposure to specific antibiotic classes—particularly cephalosporins, carbapenems, and monobactams—is associated with ROP.	Zhang et al., 2025, Patangia et al., 2022 [23,24]
Oxygen Exposure	Neonatal oxygen exposure can induce hyperoxia-driven gut dysbiosis and disrupt the natural transition of the gut from oxygen-tolerant pathogens to beneficial anaerobes [62].	Hyperoxia stalls early retinal vessel development (Phase 1 ROP) and kills protective gut anaerobes. The resulting oxygen-induced dysbiosis triggers systemic inflammation, exacerbating the subsequent pathological vessel overgrowth (Phase 2 ROP) driven by late-stage hypoxia and VEGF surges [63,64,65].	Lee 2025 [64]

## Data Availability

No new data were created or analyzed in this study. Data sharing is not applicable to this article.

## Abbreviations

The following abbreviations are used in this manuscript:

ROP	Retinopathy of prematurity
IGF-1	Insulin-like growth factor-1
VEGF	Vascular endothelial growth factor
SCFA	Short-chain fatty acid
